# Measles in Horses

**Published:** 1891-11

**Authors:** Daniel D. Lee

**Affiliations:** Instructor in Anatomy, Veterinary Department, Harvard University


### MEASLES IN HORSES.

SAME.

I have seen a number of horses, within the last two weeks, all of which were kept in the same stable. These horses were sick on an average about five days.

For the first two or three days they were feverish, refused to eat, had eyes very much inflamed, some sore throat and nasal discharge. The skin was very hot and tender, and about the third day an eruption like that seen in surfeit appeared on the skin. After this their appetite at once returned and all the other symptoms disappeared.

I thought the attack one of "pink eye," but the entire absence of any complications, and the fact that there was no prostration of strength, such as we have in most influenza cases, together with the eruption made me think differently.

No cases as yet have been seen outside this particular stable; this does not look like influenza. I find no treatment other than the removal of the grain is necessary. There have been about eighteen cases, nearly all of them acclimated horses.

